# The prognostic role of pre-treatment neutrophil to lymphocyte ratio and platelet to lymphocyte ratio in esophageal squamous cell carcinoma treated with concurrent chemoradiotherapy

**DOI:** 10.1186/s12885-024-12242-5

**Published:** 2024-04-15

**Authors:** Qirong Man, Peishun Li, Jing Fan, Sen Yang, Chao Xing, Yunling Bai, Miaomiao Hu, Baohu wang, Kaixian Zhang

**Affiliations:** Department of Oncology, Tengzhou Central People’s Hospital, Shandong, China

**Keywords:** Esophageal squamous cell carcinoma, Neutrophil, Platelet, Lymphocyte, Concurrent chemoradiotherapy

## Abstract

**Purpose:**

In this study, we retrospectively investigated the prognostic role of pre-treatment neutrophil to lymphocyte ratio (NLR) and platelet to lymphocyte ratio (PLR) in esophageal squamous cell carcinoma patients (ESCC) treated with concurrent chemo-radiotherapy (CCRT).

**Methods:**

We retrospectively analyzed the records of 338 patients with pathologically diagnosed esophageal squamous cell carcinoma that underwent concurrent chemo-radiotherapy from January 2013 to December 2017. Univariate and multivariate analyses were used to identify prognostic factors for progression free survival (PFS) and overall survival (OS).

**Results:**

The result showed that the thresholds for NLR and PLR were 2.47 and 136.0 by receiver operating characteristic curve. High NLR and PLR were both associated with tumor length (*P* < 0.05). High NLR and PLR were significantly associated with poor PFS and OS. Multivariate analyses identified NLR, PLR and TNM stage were independent risk factors for PFS and OS.

**Conclusions:**

We show that the pre-treatment NLR and PLR may serve as prognostic indicators for esophageal squamous cell carcinoma treated with concurrent chemo-radiotherapy.

## Introduction

As one of the most common malignant tumors of the digestive tract, esophageal carcinoma was reported to the sixth leading cause of cancer-related death in the world in 2018 [1]. The most common histological type is squamous cell carcinoma in Asian countries, while adenocarcinoma is more common in Western countries [2, 3]. Based on the RTOG 85–01 study, concurrent chemo-radiotherapy has been established as a curative treatment paradigm for inoperable locally advanced esophageal carcinoma [4]. Concurrent chemo-radiotherapy (CCRT) plays an important role in the treatment of esophageal carcinoma [5–8], but the overall 5-year survival rate of patients with esophageal cancer is only 15%-25% [9]. Currently, treatment options for esophageal carcinoma and disease prognosis depend mainly on the clinicopathological factors and imaging tests. However, clinical outcomes vary widely even among patients with the same clinicopathological factors. Therefore, it becomes more and more important to find novel prognostic factors that can be used for esophageal carcinoma.

As part of the tumor microenvironment, inflammation plays an important role in both the development and progression of the tumor [10–14]. Inflammatory response can be expressed by peripheral blood indicators, such as neutrophil to lymphocyte ratio (NLR) and platelet to lymphocyte ratio (PLR). Indeed, NLR and PLR have been reported to be significantly associated with the prognosis of patients with malignant tumors [15–20]. However, further studies are needed to confirm the relationship between NLR and PLR and the prognosis of thoracic esophageal squamous cell carcinoma. In some studies, high NLR and PLR have been suggested to be associated with poor prognosis, but other studies have not confirmed this association [21–24]. Therefore, the purpose of this study was to explore the value of pre-treatment peripheral blood NLR and PLR as the prognostic factors for patients with thoracic esophageal squamous cell carcinoma treated with concurrent chemo-radiotherapy.

## Materials and methods

### Patient eligibility

From January 2013 to December 2017, there were 839 patients with esophageal squamous cell carcinoma underwent CCRT in our hospital. Patients were omitted from this study if they met any of the following exclusion criteria: (a) patients with any other malignancy before or during the study; (b) patients who had received previous chemotherapy or radiotherapy; (c) patients with esophageal squamous cell cancer after surgical treatment; (d) patients who received palliative or supportive treatment only; (e) patients with infections and preoperative treatment that would alter the blood parameters; (f) patients who received certain medications within 2 weeks before blood sample collection, such as granulocyte colony-stimulating factor or thrombopoietin; (g) other serious medical conditions, such as myocardial infarction, cerebral infarction or severe cardiac insufficiency; and (f) patients without complete clinical and follow-up data. All patients were discussed by thoracic surgeons, oncologists, radiologists, and radio-physicists before deciding on a course of treatment. Through the above filters, 338 patients were selected. The retrospective research was approved by the Ethics Committee of Tengzhou Central People’s Hospital. The baseline imaging included endoscopy with biopsy, endoscopic ultrasonography (EUS), barium esophagography, cervical、chest and abdominal enhanced computed tomography (CT), and, if available, positron emission tomography-computed tomography (PET-CT).

### NLR and PLR calculation before treatment

Blood routine parameters were detected by SYSMEX-800i automatic hematology analyzer. Fasting venous blood samples were collected within 1 week before treatment, using EDTA vacuum anticoagulant tubes. Laboratory data were extracted from the electronic medical records.

From the hematological records, the NLR was calculated by dividing the absolute neutrophil count by absolute lymphocyte count.

$$NLR= \frac{Absolute Neutrophil Count}{ Absolute Lymphocyte Count}$$

The PLR was calculated by dividing the absolute platelet count by the absolute lymphocyte count.

$$PLR= \frac{Absolute P{\text{latelet}} Count}{ Absolute Lymphocyte Count}$$

### Treatment protocol

All patients were treated with concurrent chemo-radiotherapy. A total radiation dose of 50.4 -64 Gy (1.8–2.2 Gy/day, 5 days/week) was given. Limit Organ at Risk (OAR) dose: Dmean ≤ 13 Gy for both lungs, V20 ≤ 25% for both lungs, Spinal cord PRVmax ≤ 45 Gy, V30 ≤ 40% and V40 ≤ 30% for heart. The chemotherapy regimen used was TP (Paclitaxel and Cisplatin) or PF (5-Fluorouracil and Cisplatin) or S-1 (age 70 years or older). The chemotherapy was given on the first and 22nd day of RT. Two cycles of chemotherapy were completed during RT.

### Result assessment and follow-up

**Fig. 1:**
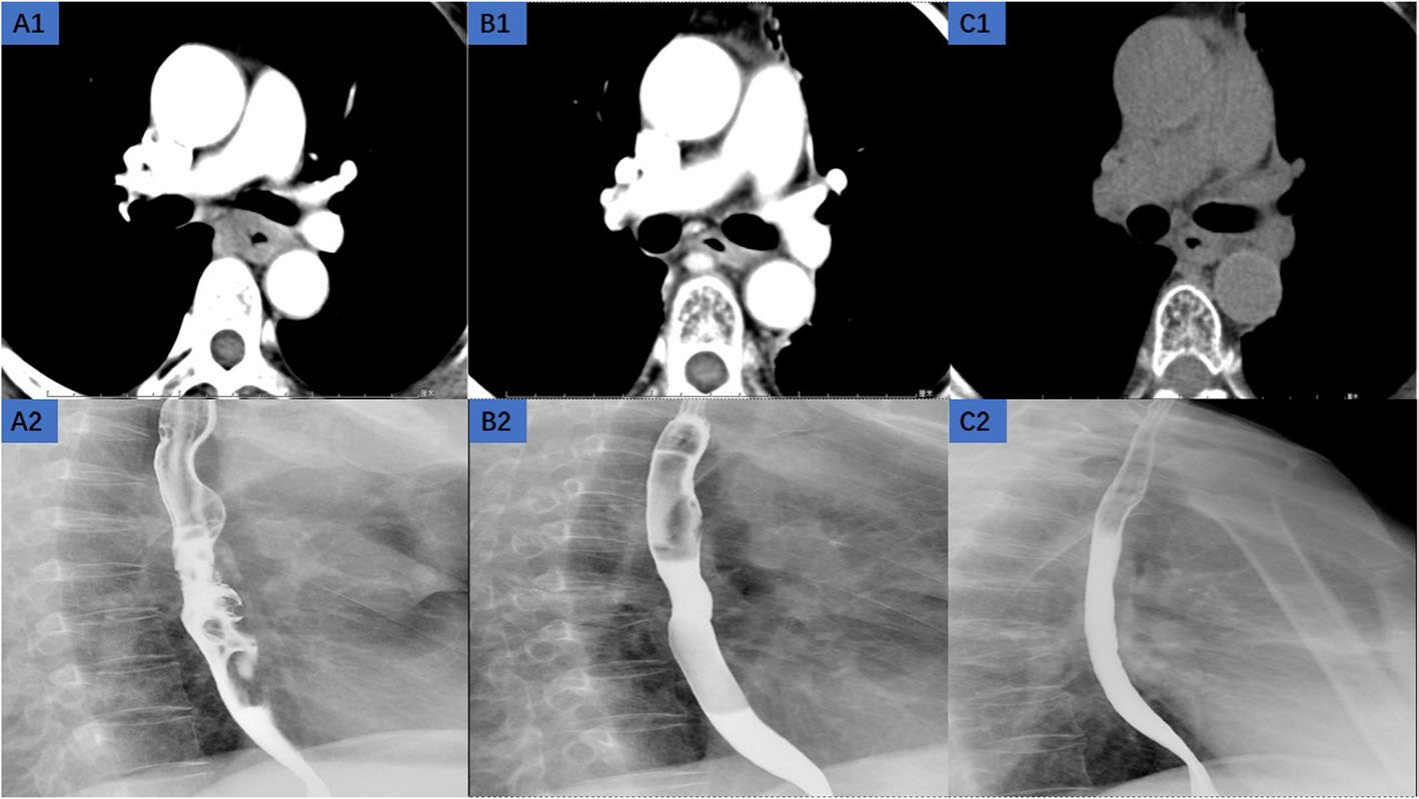
The progression-free patient. A 54-year-old woman presenting with esophageal squamous cell carcinoma, (**A**) A1: the enhanced chest CT before the treatment, A2: the esophageal barium meal imaging before the treatment; (**B**) B1: the enhanced chest CT at 6 months after the treatment, B2: the esophageal barium meal imaging at 6 months after the treatment; (**C**) C1: the enhanced chest CT at 78 months after the treatment, C2: the esophageal barium meal imaging at 78 months after the treatment

**Fig. 2:**
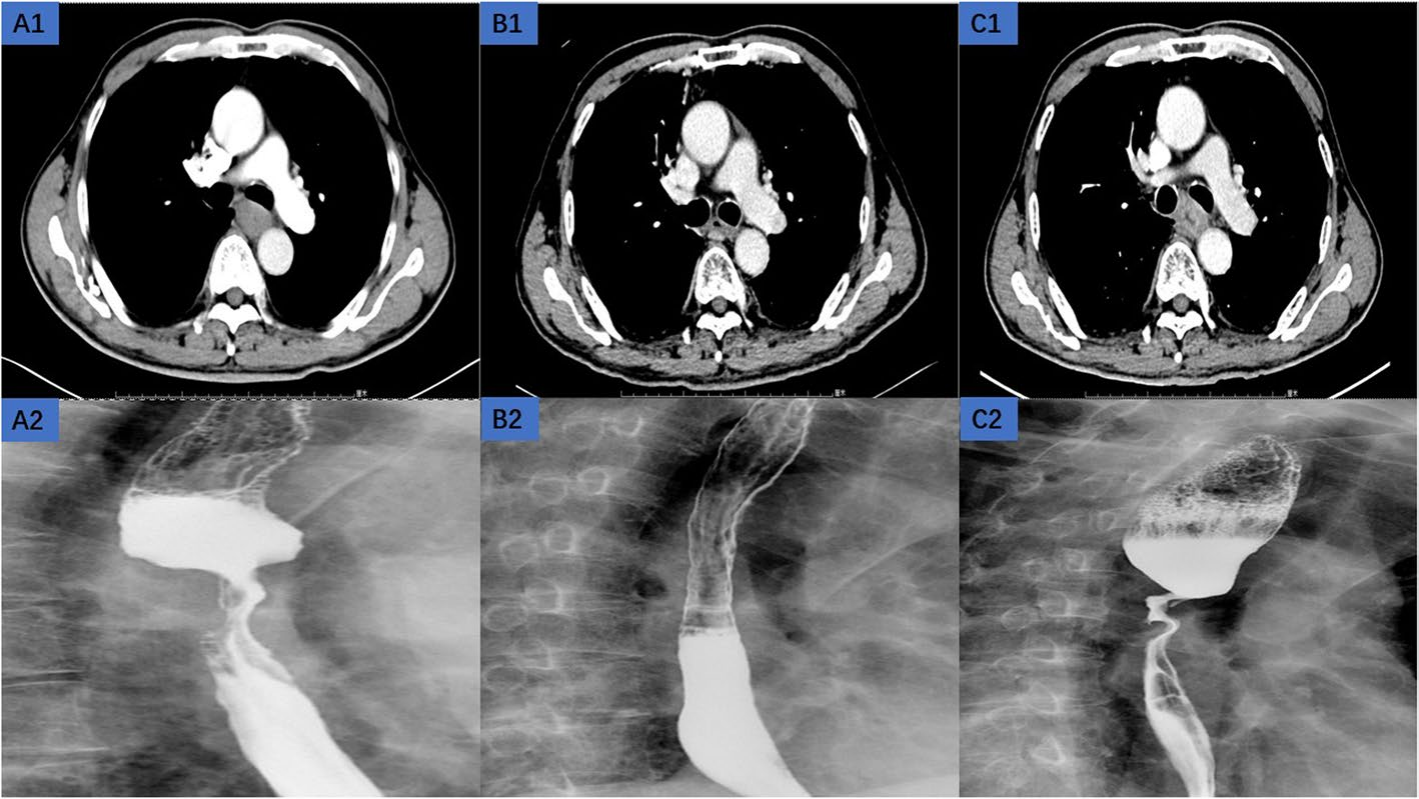
The progression patient. A 75-year-old man presenting with esophageal squamous cell carcinoma, (**A**) A1: the enhanced chest CT before the treatment, A2: the esophageal barium meal imaging before the treatment; (**B**) B1: the enhanced chest CT at 6 months after the treatment, B2: the esophageal barium meal imaging at 6 months after the treatment; (**C**) C1: the enhanced chest CT at 18 months after the treatment, C2: the esophageal barium meal imaging at 18 months after the treatment

The tumor response and recurrence were evaluated and classified according to the Response Evaluation Criteria in Solid Tumors (RECIST) system [25] and the final results were recorded by the follow-up data. In brief, the responses were classified as follows: complete response (CR), partial response (PR), progressive disease (PD), stable disease (SD). The progression-free was defined as the CR, the PR plus the SD (Fig. 1). The progression was defined as the PD (Fig. 2). All patients were followed every 3 months for the first 2 years after the end of treatment and then every 6 months until January 1, 2023 or until death. The end points of the study were PFS (Progression-Free-Survival, time from the day of treatment to progression or death from any cause during the treatment period) and OS (Overall-Survival, time from the day of treatment to January 1, 2023 or until death from any cause). Adverse events were evaluated according to the National Cancer Institute's Common Terminology Criteria for Adverse Events (CTCAE v4.03).

### Statistical analysis

The cut off values for NLR and PLR was determined using the Receiver Operator Characteristic (ROC) curve analysis. The statistical tests used were Chi square test to compare the categorical variables. Survival curves were plotted using the Kaplan–Meier method. The independent factors that are related to survival were tested using Cox regression model, forward selection was used. All statistical analyses were performed using SPSS version 23.0 (SPSS, Inc.), and statistical significance was defined as *P* < 0.05.

## Results

### Patient characteristics

**Fig. 3:**
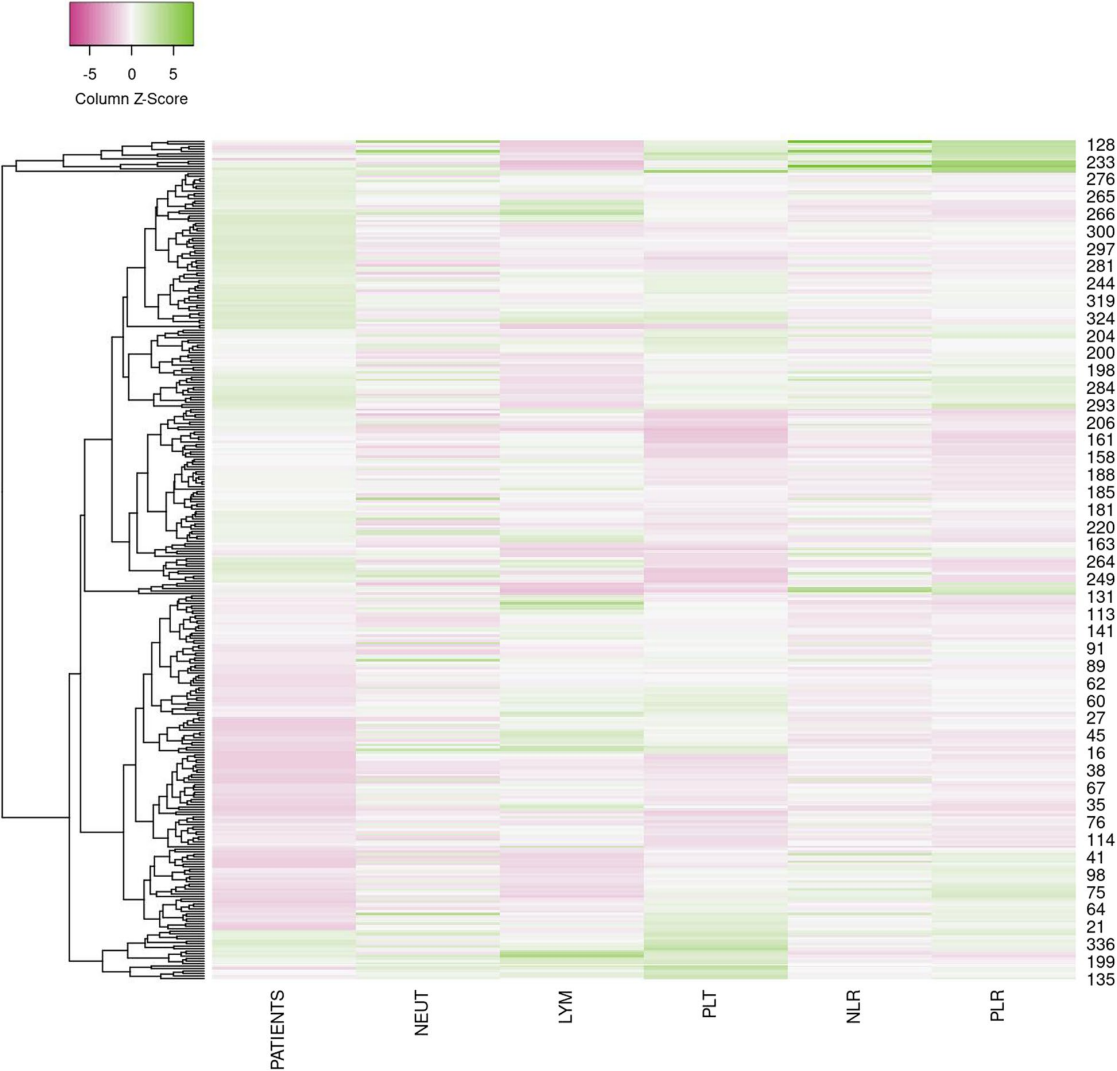
The heatmap of the original data about patients’ hematological records. NEUT: neutrophil, LYM: lymphocyte, PLT: platelet

A total of 338 patients were eligible to be included in this retrospective study. The median age at the time of diagnosis ranged from 33—76 years, with a mean age of 56.90 years for males and 57.86 years for females. 111 (32.84%) patients were females and 227 (67.16%) were males. The ratio of smokers to non-smokers was 1.06:1. The ratio of alcohol drinkers to non-drinkers was 1:1.36. The mean tumor length was 5.96 cm, and 39.05% (132/338) of the tumors were longer than 6.0 cm. All the clinicopathologic characteristics were shown in Table 1. The heatmap of the original data about patients’ hematological records was shown in Fig. 3.

**Table 1:** Clinical characteristics of 338 patients with esophageal squamous cell carcinoma

	N	%
Gender		
Male	227	67.16%
Female	111	32.84%
Age Groups (year)		
31–40	11	3.26%
41–50	72	21.30%
51–60	127	37.57%
61–70	111	32.84%
71–80	17	5.03%
Smoking history		
Yes	174	51.48%
No	164	48.52%
Alcohol history		
Yes	143	42.31%
No	195	57.69%
Clinical TNM classification		
Stage I-II	97	28.70%
Stage III-IV	241	71.30%
Tumor length (cm), mean (± SD)	5.96 (± 2.03)	
Tumor location		
Upper thoracic	95	28.11%
Middle thoracic	167	49.41%
Lower thoracic	76	22.48%
Adverse event		
Grade ≥ 3	36	10.65%
Grade < 3	302	89.35%

### The ROC Curve for Optimal Cutoff Value and AUC

**Fig. 4:**
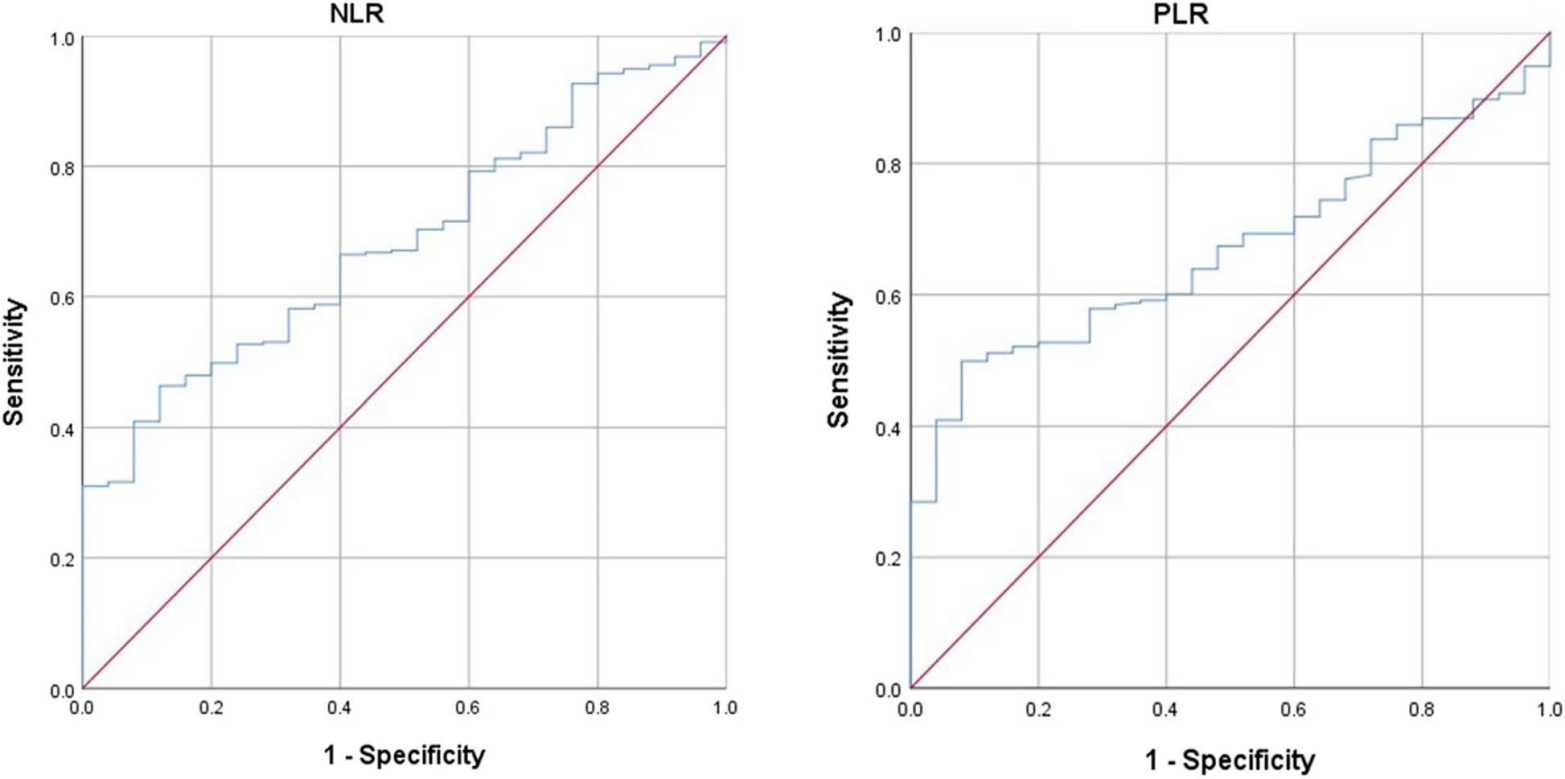
ROC curves for NLR and PLR

The ROC curves are plotting as Fig. 4. When the NLR was 2.47, YI index was at its maximum (YI = 0.343), demonstrating that 2.47 was the optimal cutoff value for NLR (AUC = 0.686 with the sensitivity of 46.3% and specificity 88.0%, *P* = 0.002). Therefore, patients were divided into low NLR (< 2.47) and high NLR (≥ 2.47) groups. Similarly, When the PLR was 136, YI was at its maximum (YI = 0.418), demonstrating that 136 was the optimal cutoff value for PLR (AUC = 0.670 with the sensitivity of 49.8% and a specificity 92.2%, *P* = 0.005). Therefore, patients were divided into low PLR (< 136) and high PLR (≥ 136) groups.

### Association of peripheral blood cell count ratios with clinical characteristics

All the clinicopathologic characteristics were comparable between patients grouped by NLR or PLR, as shown in Table 2. There were significant differences in gender, TNM Stage and tumor length among different NLR groups (all *P* < 0.05). There were significant differences in smoking and tumor length among different PLR groups (all *P* < 0.05).

**Table 2:** Clinicopathological Characteristics between patients grouped by NLR or PLR

Characteristics	NLR			PLR		
	NLR < 2.47 (Number, %)	NLR ≥ 2.47 (Number, %)	p	PLR < 136 (Number, %)	PLR ≥ 136 (Number, %)	p
Total Gender	191(100)	147(100)		172(100)	166(100)	
Male	109(57.1)	118(80.3)	0.000	117(68)	110(66.3)	0.731
Female	82(42.9)	29(19.7)		55(32)	56(33.7)	
Age (year)						
< 65	155(81.2)	114(77.6)	0.416	141(82)	128(77.1)	0.267
≥ 65	36(18.8)	33(22.4)		31(18)	38(22.9)	
Smoking						
Yes	92(48.2)	82(55.8)	0.165	99(57.6)	75(45.2)	0.023
No	99(51.8)	65(44.2)		73(42.4)	91(54.8)	
Alcohol						
Yes	73(38.2)	70(47.6)	0.083	71(41.3)	72(43.4)	0.697
No	118(61.8)	77(52.4)		101(58.7)	94(56.6)	
TNM Stage						
Stage I-II	64(33.5%)	33(22.4%)	0.026	51(29.7%)	46(27.7%)	0.693
Stage III-IV	127(66.5%)	114(77.6%)		121(70.3%)	120(72.3%)	
Tumor length						
< 5 cm	68(35.6%)	30(20.4%)	0.002	60(34.9%)	38(22.9%)	0.015
≥ 5 cm	123(64.4%)	117(79.4%)		112(65.1%)	128(77.1%)	
Tumor location						
Upper thoracic	62(32.5%)	33(22.4%)	0.061	56(32.6%)	39(23.5%)	0.160
Middle thoracic	93(48.7%)	74(50.3%)		78(45.3%)	89(53.6%)	
Lower thoracic	36(18.8%)	40(27.3%)		38(22.1%)	38(22.9%)	

### Survival analysis

**Fig. 5:**
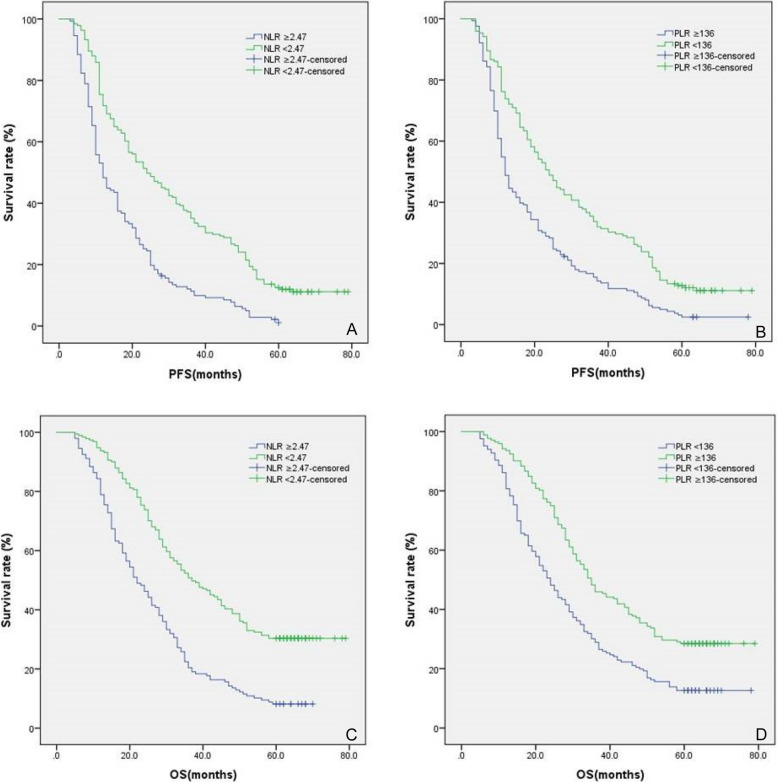
Kaplan–Meier plots of PFS and OS according to NLR and PLR. **A** NLR ≥ 2.47 was an adverse prognostic factor for PFS (*P* < 0.001). **B** PLR ≥ 136 was an adverse prognostic factor for PFS (*P* < 0.001). **C** NLR ≥ 2.47 was an adverse prognostic factor for OS (*P* < 0.001). (D) PLR ≥ 136 was an adverse prognostic factor for OS (*P* < 0.001)

Kaplan-Mier survival curve analysis showed that the 3-year PFS of patients in the high NLR group and the low NLR group were 11.4.0% and 37.2%, respectively (*P* < 0.05; Fig. 5A). The 3-year PFS of the high PLR group and the low PLR group were 15.5% and 33.7%, respectively (*P* < 0.05; Fig. 5B).

Kaplan-Mier survival curve analysis showed that the 5-year OS of 8.2% in the high NLR group was significantly lower than 30.4% in the low NLR group (*P* < 0.05, Fig. 5C). The 5-year OS of the high PLR group was 12.7%, which was significantly lower than 28.5% of the low PLR group (*P* < 0.05, Fig. 5D).

### Relationship between elevated NLR or PLR and prognosis

#### For PFS

In the univariate analysis, we found that NLR, PLR, TNM stage and tumor length were significant factors for PFS (all *P* < 0.05) (Table 3). Then, the variables mentioned above were included in a multivariate Cox regression model for subsequent analysis. We finally demonstrated that NLR, PLR and TNM stage were independent risk factors for PFS (Table 4).

**Table 3:** Univariate analysis of factors associated with PFS and OS

Variable	PFS(months)			OS(months)		
	mPFS	95% CI	p	mOS	95% CI	p
NLR						
< 2.47	24.0	18.459–29.541	0.000	37	30.551–43.449	0.000
≥ 2.47	12.0	9.773–14.227		22	18.040–25.960	
PLR						
< 136	24.0	20.145–27.835	0.000	35	30.268–39.732	0.000
≥ 136	12.0	10.597–13.403		24	20.708–27.292	
Gender						
Male	18	15.163–20.837	0.111	29	25.720–32.280	0.089
Female	19	14.151–23.849		31	25.839–36.161	
Age (year)						
< 65	18	14.786–21.214	0.517	29	25.556–32.444	0.272
≥ 65	18	14.512–21.488		30	26.300–33.700	
Smoking						
Yes	16	11.478–20.522	0.695	28	23.077–32.923	0.613
No	19	16.718–21.282		31	28.015–33.985	
Alcohol						
Yes	16	11.614–20.386	0.407	28	22.492–33.508	0.300
No	19	16.720–21.280		30	27.149–32. 851	
TNM Stage						
Stage I-II	25	18.574–31.426	0.000	37	28.095–45.905	0.000
Stage III-IV	15	12.392–17.608		27	24.546–29.454	
Tumor length						
< 5 cm	24	19.842–28.158	0.016	34	30.767–37.233	0.032
≥ 5 cm	15	12.321–17.679		27	24.382–29.618	
Tumor location						
Upper thoracic	19	14.224–23.776	0.800	33	26.632–39.368	0.455
Middle thoracic	16	10.668–21.332		30	25.397–34.603	
Lower thoracic	17	14.670–19.330		28	26.102–29. 898

**Table 4:** Multivariate analysis of PFS

Prognostic factors	Progression-Free-Survival		
	Odds ratio	95% CI	p
NLR	0.575	0.449–0.737	0.000
PLR	0.677	0.531–0.863	0.002
TNM stage	1.427	1.102–1.849	0.007
Tumor length	0.924	0.715–1.194	0.544

#### For OS

In the univariate analysis, we found that NLR, PLR, TNM stage and tumor length were significant factors for OS (all *P* < 0.050) (Table 3). Then, the variables mentioned above were included in a multivariate Cox regression model for subsequent analysis. We finally demonstrated that NLR, PLR and TNM stage were independent risk factors for OS (Table 5).

**Table 5:** Multivariate analysis of OS

Prognostic factors	Overall-Survival		
	Odds ratio	95% CI	p
NLR	0.539	0.414–0.701	0.000
PLR	0.712	0.548–0.925	0.011
TNM stage	1.481	1.113–1.971	0.007
Tumor length	0.960	0.726–1.271	0.777

## Discussion

As is reported, neutrophils, platelets, and lymphocytes participate a lot in the tumor‑related inflammation and immunology [26]. Various authors have investigated the role of NLR and PLR in many malignancies [27–30] and found that a higher NLR and PLR are associated with worse prognosis in terms of recurrence and mortality.

In this study, the threshold value of NLR and PLR was obtained by using the Receiver Operator Characteristic (ROC) curve, and then the pre-treatment NLR and PLR were assessed in 338 patients with ESCC and correlated with demographic data and tumour characteristics. There were significant differences in gender, TNM Stage and tumor length among different NLR groups (all P < 0.05). There were significant differences in smoking and tumor length among different PLR groups (all *P* < 0.05) (Table 2). In contrast to our study, Chen et al., Acharya et al., and Nakashima et al., found no statistical significance with age and gender with a high PLR and elevated NLR, respectively [31–33]. However, Xie et al. [34] found that NLR level was different in gender, lymph node metastasis and tumor length, and PLR level was different in tumor length and tumor stage, and pointed out that PLR level could play a predictive role in the prognosis of patients with esophageal cancer stage I-II. This conclusion is similar to our study. The differences in the results of the above studies may be caused by the specificity of study region and population, the difference in the selection of NLR and PLR cut-off points, and the limitation of sample size.

Neutrophils, in addition to the production of angiogenic cytokines like: vascular endothelial growth factor and interlukin-8 (IL-8), they also produce matrix metalloprotein-9 (MMP-9) (gelatinase B) that induces an angiogenic state in the tumour cells. Neutrophil gelatinase-associated lipocalin is linked to the depth of invasion and tumour differentiation. Apart from neutrophils, the tumour associated macrophages and other leukocytes have been related with aggressive tumour behaviour of invasion and metastasis through MMP-2 by inducing extracellular matrix remodeling [35]. Therefore, a high pre-treatment neutrophil–lymphocyte ratio (NLR) may represent an underlying favourable microenvironment for the growth of tumour cells and aggressiveness of the tumour, favouring its spread/micro-metastasis and ultimately recurrence.

Sato et al. [36] demonstrated that NLR can be used as a new prognostic predictor for patients with thoracic esophageal cancer treated with neo-adjuvant chemotherapy (cisplatin and 5-fluorouracil). In this study, peripheral blood NLR was calculated in 83 patients with esophageal cancer who received preoperative chemotherapy with the above regimens, and the pathological response of the tumor after chemotherapy was compared. The results showed that the pathological response rate of patients with NLR ≥ 2.2 before chemotherapy was 21%, while that of patients with NLR < 2.2 was 56%. Elevated NLR (NLR ≥ 2.2) in peripheral blood before chemotherapy predicts lymph node metastasis and low pathological response rate. Sharaiha et al. [37] retrospectively collected 295 patients with esophageal cancer who underwent radical resection from 1996 to 2009 to explore the relationship between preoperative NLR level and prognosis. The overall survival (OS) (P = 0.0002) and disease-free survival (DFS) (P < 0.0001) were significantly different between patients with a high NLR level and those with a low NLR level. Patients with elevated preoperative NLR (NLR ≥ 5.0) had lower OS and DFS. Univariate analysis in our study showed that both progression free survival and overall survival were worse in the high NLR group than in the low NLR group. Multivariate analysis showed that pre-treatment NLR was an independent prognostic factor for postoperative recurrence and death in patients with esophageal cancer.

Feng et al. [38] recorded and calculated the PLR of 483 patients with esophageal cancer, with 150 as the cut-off point, and recorded and calculated the preoperative NLR value, which was divided into two groups with 3.5 as the cut-off point, in order to compare the relationship between the two indexes and the OS of the two groups. The results showed that the OS of patients with high PLR level was significantly different from that of patients with low PLR level (32.7% vs 63.5%, P < 0.001). Moreover, high PLR level was associated with poor OS and DFS (P < 0.001). Univariate analysis in our study showed that both progression free survival and overall survival were worse in the high PLR group than in the low PLR group. Multivariate analysis showed that pre-treatment PLR was an independent prognostic factor for postoperative recurrence and death in patients with esophageal cancer.

Our study also found that PLR is not related to TNM stage which is strongly closed to survival. The reason for this interesting phenomenon may be: (1) PLR offers simple, readily available measures of systemic inflammation, which is associated with a variety of causes, including disorders of the blood system, inflammation, and drug side effects, rather than directly related to the tumor itself;(2) TNM staging is determined based on tumor size, location, whether it has spread or metastasized, and the extent of such spread or metastasis. Therefore, there is no direct relationship between platelet to lymphocyte ratio and tumor TNM stage; however, this does not mean that platelet to lymphocyte ratio has no value in the treatment of cancer patients. For example, Messageri et al. [39] explored the relationship between PLR and the prognosis of patients with esophageal cancer after neo-adjuvant chemotherapy, and collected the data of patients with esophageal cancer or esophago-gastric junction cancer. They found a difference in recurrence rate between patients with high PLR levels and those with low PLR levels (54.8% v 35.2%, *P* = 0.046). Moreover, high PLR level was associated with poor OS and DFS (*P* < 0.001). This is similar to the results of our study.

The possible explanations for high PLR to be associated with poorer prognosis are that platelets can encourage tumour growth by increasing angiogenesis and also increase the microvascular permeability leading to extravasation of the tumour cells into the circulation [40–42]. Secondly, they interact with tumour cells via ligands or receptors and help in growth of the tumour or invasion [43, 44]. Thirdly, it has been proposed that platelets protect the tumour cells from the natural killer (NK) cells, by reducing their cytolytic activity [45]. Moreover, patients who have a high PLR also show relative lymphocytopenia, which may be responsible for inadequate immune system leading to weakened defence against cancer [31, 46, 47].

This study differs from other studies in the following areas: (1) This study focused on patients with esophageal squamous cell carcinoma who received concurrent chemoradiotherapy; (2) Multivariate analysis was used to explore the combined effect of NLR and PLR with other prognostic factors; (3) The results of this study showed that NLR/PLR was significantly associated with the prognosis of patients, which helps clinicians to provide a new reference for the treatment decision of patients with esophageal squamous cell carcinoma.

But our study has several limitations. First of all, it is a retrospective study including only one institution, which may lead to a selection bias. Second, with limited data availability, we did not study other inflammatory biomarkers, such as C-reactive protein and the lymphocyte to monocyte ratio. Third, the long data collection time in this retrospective analysis and advances in concurrent chemo-radiotherapy during this period may influence the clinical outcome. All patients in this study had squamous cell carcinoma, so the results may not be generalizable to adenocarcinoma. At the same time, all patients in our study were Asians, and whether the results of this study can be generalized to white and black population needs further study. Finally, the findings from this study can only provide prognostic significance about tumor behavior and can not yet be used to alter current treatment recommendation. Therefore, a multicenter collaborative study is required to be further verified in a prospective, large-scale collaborative study.

NLR and PLR may offer a simple and widely available laboratory test to evaluate the prognosis of thoracic esophageal squamous cell carcinoma with concurrent radiotherapy and chemotherapy. By predicting which patients will have a favorable treatment response, clinicians may be able to individualize cancer treatment plans to avoid over or undertreating patients. Evaluating NLR and PLR may prove useful in future recommendations for individualized esophageal cancer care.

## Data Availability

The data that support the findings of this study are not openly available due to reasons of sensitivity and are available from the corresponding author upon reasonable request.
